# Intercomparison of phenological transition dates derived from the PhenoCam Dataset V1.0 and MODIS satellite remote sensing

**DOI:** 10.1038/s41598-018-23804-6

**Published:** 2018-04-09

**Authors:** Andrew D. Richardson, Koen Hufkens, Tom Milliman, Steve Frolking

**Affiliations:** 1 Northern Arizona University, School of Informatics, Computing and Cyber Systems, Flagstaff, AZ 86011 USA; 2 Northern Arizona University, Center for Ecosystem Science and Society, Flagstaff, AZ 86011 USA; 3 INRA, UMR ISPA, Villenave d’Ornon, France; 4 University of New Hampshire, Earth Systems Research Center, Durham, NH 03824 USA

## Abstract

Phenology is a valuable diagnostic of ecosystem health, and has applications to environmental monitoring and management. Here, we conduct an intercomparison analysis using phenological transition dates derived from near-surface PhenoCam imagery and MODIS satellite remote sensing. We used approximately 600 site-years of data, from 128 camera sites covering a wide range of vegetation types and climate zones. During both “greenness rising” and “greenness falling” transition phases, we found generally good agreement between PhenoCam and MODIS transition dates for agricultural, deciduous forest, and grassland sites, provided that the vegetation in the camera field of view was representative of the broader landscape. The correlation between PhenoCam and MODIS transition dates was poor for evergreen forest sites. We discuss potential reasons (including sub-pixel spatial heterogeneity, flexibility of the transition date extraction method, vegetation index sensitivity in evergreen systems, and PhenoCam geolocation uncertainty) for varying agreement between time series of vegetation indices derived from PhenoCam and MODIS imagery. This analysis increases our confidence in the ability of satellite remote sensing to accurately characterize seasonal dynamics in a range of ecosystems, and provides a basis for interpreting those dynamics in the context of tangible phenological changes occurring on the ground.

## Introduction

Phenology is a key driver of the seasonality of ecosystem processes, an essential indicator of the biological impacts of climate change, and a valuable diagnostic of ecosystem health for land managers^1^. However, observer records of phenology are inherently subjective. And, it is difficult to make these observations at sufficiently high temporal resolution, and sufficiently dense spatial sampling, to fully characterize phenological patterns at ecosystem (or larger) scales. While satellite remote sensing of phenology provides global information on the seasonality of vegetation activity^2,3^, tradeoffs between spatial and temporal resolution—not to mention data quality issues associated with atmospheric corrections, cloud screening, and varying look angles—are also acknowledged to result in substantial uncertainties^4^. Multi-scale monitoring of vegetation phenology, which requires linking observations at the scale of individual organisms to patterns recorded via satellite remote sensing at the ecosystem or regional scale, can potentially overcome some of these limitations and therefore help to improve resource management and decision-making^5,6^. Real-time phenological forecasting, leveraging this multi-scale perspective, would further contribute to such efforts^7^.

However, progress towards these objectives is hindered by uncertainties regarding the congruence between what is happening on the ground and what is actually observed by satellite sensors. And, more generally, the lack of standardized phenological data sets, with scale-relevant observations, has—until recently—hobbled the evaluation and interpretation of remotely sensed phenological data products^8^.

Over the last two decades, technological developments have reduced the cost and the infrastructure required to develop on-the-ground monitoring networks that can provide data on vegetation phenology in real time and at high spatial and temporal resolution^9^. The data from this near-surface remote sensing, whether derived from radiometric instruments^10,11^ or imaging sensors^12–14^, is critical for improving our understanding of the strengths and limitations satellite remote sensing of phenology, and for conducting cross-scale phenological data integration^5^.

The PhenoCam Network is one such near-surface monitoring effort. PhenoCam uses imagery from digital cameras to track vegetation phenology at high temporal resolution^12,15,16^. Images, recorded every 30 minutes from dawn to dusk, are processed using analysis methods that yield quantitative information about changes in vegetation color, from which measures of phenology can be derived in a manner that is analogous to the processing of vegetation indices derived from satellite imagery^17^. With a canopy-scale perspective, but the capacity to resolve individual organisms, the PhenoCam approach therefore provides a direct link between on-the-ground monitoring by human observers and satellite-based monitoring at comparatively coarse spatial and temporal resolution^18^.

At present, over 400 cameras are contributing imagery to PhenoCam; sites are distributed across North America, from Alaska to Florida and from Hawaii to Arizona to Maine, with additional sites in Central and South America, and Europe^12^. A number of previous studies have used relatively small subsets of PhenoCam data to evaluate vegetation phenology derived from the MODIS^17,19–22^, Landsat^21,23,24^, AVHRR^17^, VIIRS^22,25^, and MERIS^26^ platforms. Most of these studies have focused on temperate and boreal deciduous forests, with little attention to other ecosystems or vegetation types.

Efforts to produce a curated, processed and quality-controlled dataset for public release have resulted in the PhenoCam Dataset V1.0^27^, which consists of nearly 750 site-years of observations from the tropics to the subarctic. Time series of “canopy greenness” have been processed to yield phenological transition dates corresponding to the start of the “greenness rising” and end of the “greenness falling” cycles of vegetation activity^12^. This data set offers a unique opportunity to investigate the spatiotemporal coherence between phenological transitions on the ground and as seen from space, and to explore the conditions associated with varying degrees of agreement.

Here we use phenological transition date data from the PhenoCam Dataset V1.0, together with MODIS dates^3^ corresponding to the timing of “greenup onset” and “dormancy onset” for the same ground location, to investigate the following questions:

1. Across all vegetation types, what is the overall agreement between phenological transitions derived from near-surface remote sensing and satellite remote sensing?
2. Do the results from (1) vary according to vegetation type, and how important is it that the vegetation in the camera field of view be representative of the larger MODIS pixel?

We conclude by discussing specific examples, with varying levels of agreement between time series of PhenoCam canopy greenness and MODIS NDVI (normalized difference vegetation index), to provide more general insight into the limitations of either approach, and to uncover potential avenues for improvement.

## Results

### Geographic and eco-climatic distribution of sites

**Figure 1:**
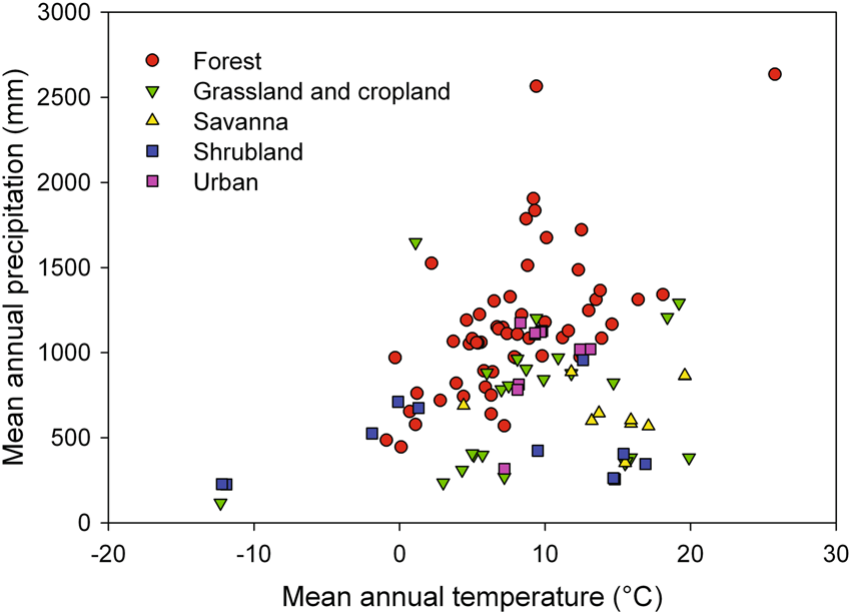
Climate space spanned by the 128 camera sites included in this analysis. Mean annual temperature and mean annual precipitation are from the WorldClim database. Symbols are colored according to a simplified IGBP land cover classification (Forest = IGBP 1, 2, 4, 5; Grassland and cropland = IGBP 10, 12, 14; Savanna = IGBP 8, 9; Shrubland = IGBP 7; Urban = IGBP 13).

We used data from 128 camera sites in our analysis (Supplementary Table 1; see also the interactive map and data visualization tools available at http://explore.phenocam.us). The average image time series was over 5 y in length. Camera sites included in our analysis range across more than 60 degrees of latitude, from 9°N to 71°N, with half of all sites falling within the latitudinal band from 40–48°N. Based on WorldClim^28^ data, these sites span almost 40 °C in mean annual temperature (from −12 °C to +26 °C; mean ± 1 SD = 8.9 ± 6.0 °C), and a 20-fold range in annual precipitation (from 115 mm to 2600 mm; mean ± 1 SD = 925 ± 435 mm) (Fig. 1).

Within the camera imagery, the dominant vegetation type was DB (deciduous broadleaf; 67 sites), followed by GR (grassland; 25 sites), EN (evergreen needleleaf; 18 sites), SH (shrub; 11 sites), and AG (agriculture, 10 sites). For 16 of 128 cameras, there were separate regions of interest (ROIs) defined for two distinct vegetation types within the camera field of view. According to MODIS land cover classification, half the camera sites were dominated by forest, with mixed forest (32 sites) and deciduous broadleaf forest (22 sites) being the most common (Table 1). Across all camera sites and ROIs, there were 581 greenness rising phenological transition dates with corresponding MODIS greenup onset retrievals, and 602 greenness falling phenological transition dates with corresponding MODIS dormancy onset retrievals.

**Table 1:** Distribution of 128 camera sites included in this analysis, according to MODIS land cover classification.

IGBP Numeric Class	Description	Count
1	Evergreen Needleleaf forest	9
2	Evergreen Broadleaf forest	1
4	Deciduous Broadleaf forest	22
5	Mixed forest	32
7	Open shrublands	11
8	Woody savannas	7
9	Savannas	3
10	Grasslands	10
11	Permanent wetlands	1
12	Croplands	14
13	Urban and built-up	10
14	Cropland/Natural vegetation mosaic	7
N/A	Not classified	1

### Overall agreement between PhenoCam and MODIS transition dates

Considering all vegetation types together, the mean greenness rising transition date calculated from our canopy greenness index, the Green Chromatic Coordinate (*G*_cc__best; see Methods), was DOY 109 ± 34 d (mean ± 1 SD, across all camera sites, ROIs, and years) for the 10% amplitude threshold, DOY 117 ± 32 d for the 25% amplitude threshold, and DOY 126 ± 31 d for the 50% amplitude threshold. These dates were all biased late relative to the mean MODIS greenup onset date of DOY 102 ± 36. Overall, the bias was 8 ± 21 day for the 10% amplitude threshold, 15 ± 20 d for 25% amplitude threshold, and 23 ± 20 d for the 50% amplitude threshold. The linear correlation between the 10% amplitude threshold date and the MODIS greenup onset date was *r* = 0.81.

By comparison, across all camera sites and vegetation types, the mean greenness falling date calculated from *G*_cc__best was 267 ± 36 d (mean ± 1 SD, across all camera sites and years) for the 50% amplitude threshold, 282 ± 36 d for the 25% amplitude threshold, and 290 ± 36 d for the 10% amplitude threshold. These dates were biased early relative to the mean MODIS dormancy onset date of DOY 298 ± 35 d. Overall, the bias was 29 ± 24 d for the 50% amplitude threshold, 16 ± 24 d for the 25% amplitude threshold, and 8 ± 25 d for the 10% amplitude threshold. The linear correlation between the 10% amplitude threshold date and the MODIS dormancy onset date was *r* = 0.74.

### Agreement between PhenoCam and MODIS transition dates, by vegetation type

In the above analysis, we lumped all vegetation types together, and did not consider whether the vegetation in the PhenoCam field of view was similar to the broader landscape as seen from MODIS. However, it is possible that the agreement between PhenoCam and MODIS is stronger for some vegetation or land cover types than others. Additionally, for some camera sites, landscape heterogeneity may result in “apples to oranges” PhenoCam-MODIS comparisons. In other words, the vegetation within the PhenoCam field of view (or for a particular PhenoCam ROI) may be representative (“apples to apples” comparison) or may not be representative (“apples to oranges” comparison) of the vegetation that is dominant at the scale of 500 m MODIS observations.

Our operating hypothesis is that by accounting for representativeness and heterogeneity, we may be able to obtain improved agreement between PhenoCam and MODIS phenological transition dates. In fact, in some but not all cases, the agreement between PhenoCam and MODIS phenology dates was indeed better for “apples to apples” comparisons than “apples to oranges” comparisons. For example, for PhenoCam DB (deciduous broadleaf) vegetation ROIs, the agreement with MODIS dates was very strong during the greenness rising phase when the MODIS pixel was assigned IGBP landcover class 4 (deciduous broadleaf forest) or 5 (mixed forest), but less strong for landcover class 13 (urban) (Supplementary Table 2). By comparison, for EN (evergreen needleleaf) vegetation ROIs, the agreement with MODIS was surprisingly poor even when the MODIS pixel was assigned IGBP landcover class 1 (evergreen needleleaf forest) or 5 (mixed forest) (Supplementary Table 2). Clearly the level of agreement between PhenoCam and MODIS depends on both the type of vegetation within the camera field of view, and the representativeness of that field of view of the broader landscape. For four vegetation types (AG, DB, EN, and GR), there were enough data (*n* ≥ 25 paired PhenoCam-MODIS observations) to examine these patterns in greater detail (Table 2).

**Table 2:** Statistics of agreement between phenological transition dates derived from PhenoCam imagery (10% seasonal amplitude threshold during “greenness rising” and “greenness falling” phenological phases) and from MODIS satellite remote sensing (onset of greenness, onset of senescence)

PhenoCam ROI Vegetation Type	IGBP Landcover	*n*	SD (camera DOY)	SD (MODIS DOY)	Pearson’s *r*	∆ DOY ± SD	Deming regression		
							Distance	Slope ± SE	Intercept ± SE
**(a) “Greenness rising” phase**									
AG	12, 14	40	65	58	0.91	−25.1 ± 27.1	18.7	0.89 ± 0.09	−12.8 ± 11.3
	Other	2	12	0		−8.5 ± 12.0			
DB	4, 5	284	15	16	0.83	−9.4 ± 9.1	6.4	1.07 ± 0.06	−17.4 ± 6.8
	Other	74	25	29	0.74	−18.0 ± 19.8	13.7	1.22 ± 0.22	−41.6 ± 24.4
EN	1, 5	49	14	15	0.37	26.1 ± 15.9	11.3	1.15 ± 0.36	13.0 ± 31.7
	Other	7	7	18	0.55	16.6 ± 15.3	6.2		
GR	10, 12, 14	49	58	60	0.97	−11.4 ± 14.5	10.3	1.03 ± 0.06	−14.7 ± 6.5
	Other	37	54	58	0.90	−6.0 ± 25.0	17.6	1.08 ± 0.12	−12.7 ± 12.1
**(b) “Greenness falling” phase**									
AG	12, 14	40	51	56	0.87	10.6 ± 27.4	19.3	1.11 ± 0.11	−17.9 ± 31.0
	Other	4	16	32	−0.42	29.8 ± 41.5	20.1		
DB	4, 5	302	12	17	0.66	15.0 ± 12.9	8.0	1.68 ± 0.18	−182.4 ± 52.7
	Other	75	33	41	0.75	4.9 ± 27.1	18.3	1.31 ± 0.22	−89.5 ± 68.0
EN	1, 5	50	23	17	0.38	−33.4 ± 22.7	14.9	0.49 ± 0.22	134.4 ± 72.3
	Other	7	13	14	0.22	−42.6 ± 16.5	12.8		
GR	10, 12, 14	50	43	47	0.84	15.4 ± 25.5	17.9	1.13 ± 0.10	−18.1 ± 26.6
	Other	36	67	64	0.89	16.6 ± 30.2	21.6	0.95 ± 0.06	30.1 ± 14.4

Data reported only for vegetation types for which there were *n* = 25 or more paired PhenoCam-MODIS observations with similar land cover classification (“apples to apples” comparisons; see text) during both greenness rising and greenness falling phases (see Supplementary Table 2 for the full [vegetation type x IGBP landcover] matrix). Vegetation types are as follows: AG = agricultural; DB = deciduous broadleaf; EN = evergreen needleleaf; GR = grassland. IGBP Landcover classes are defined in Table 1. ROI = region of interest; SD = standard deviation; SE = standard error; DOY = day of year. Deming (orthogonal) regression slope and intercept reported only when the slope is significantly different from 0 at *p* < 0.05 and *n* ≥ 10. The Deming regression distance is the RMS (root mean squared) distance, measured perpendicular to the regression line and assuming (*n* − 2) degrees of freedom.

**Figure 2:**
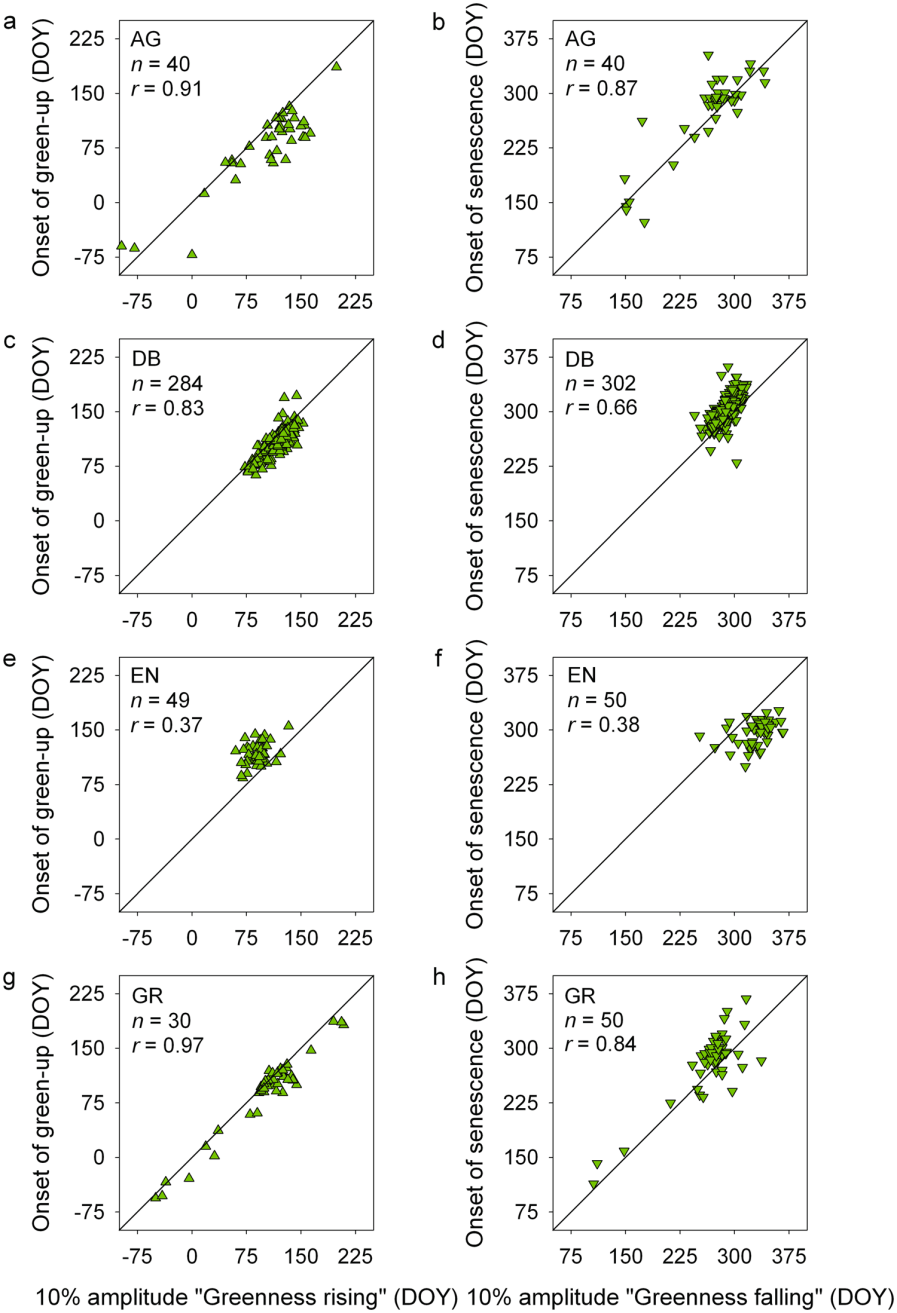
Pairwise correlation between phenological transition dates derived from PhenoCam imagery and MODIS satellite remote sensing. Results are broken down by vegetation type: (**a**,**b**) agriculture (AG); (**c**,**d**) deciduous broadleaf forest (DB); (**e**,**f**) evergreen needleleaf forest (EN); and (**g**,**h**) grassland (GR). For clarity, PhenoCam greenness rising (*x*) and MODIS onset of green-up (*y*) transition dates (left column) after DOY 270 have been wrapped to the beginning of the year (DOY < 0), while PhenoCam greenness falling (*x*) and MODIS onset of senescence (*y*) transition dates (right column) prior to DOY 90 have been wrapped to the end of the year (DOY > 365).

For AG sites, the “apples to apples” correlation between PhenoCam and MODIS was generally excellent (Table 2, Fig. 2), although MODIS dates tended to be biased early by almost a month during the greenness rising phase, and biased late by a smaller amount during the greenness falling phase (Table 2). However, Deming regression slopes were not significantly different from 1.0 in either phase (Table 2). Based on the perpendicular regression distance *d*, the mean regression error was less than three weeks, although there were several instances of much larger *d* (six weeks or more) (Table 2). There were not sufficient data to make a meaningful assessment of “apples to oranges” comparisons for AG sites.

For DB sites, the “apples to apples” agreement between PhenoCam and MODIS was very good overall (Fig. 2). Unlike the AG sites, large discrepancies between PhenoCam and MODIS dates were rare for the DB sites. But, similar to AG sites, MODIS dates were again biased early during the greenness rising phase, and biased late during the greenness falling phase (Table 2). The Deming regression slope was indistinguishable from one during the greenness rising phase, but was significantly greater than one during the greenness falling phase (Table 2). For “apples to apples” comparisons, regression distances *d* were substantially lower for DB sites than for AG, EN, or GR (Table 2). However, for “apples to oranges” comparisons, regression distances *d* were roughly twice as high as for “apples to apples” comparisons, and thus similar to those for other vegetation types (Table 2).

For EN sites, low correlation coefficients during both greenness rising and falling phases indicate the relatively poor agreement between PhenoCam and MODIS dates (Fig. 2), even for “apples to apples” comparisons (Table 2). In contrast to AG and DB sites, MODIS dates were biased late during the greenness rising phase, but biased early by an even larger amount during the greenness falling phase (Table 2). Indeed, it is readily apparent (Fig. 2) that the PhenoCam–MODIS agreement is much worse for evergreen conifers than other vegetation types. Note that this result is not driven by the inclusion of IGBP landcover class 5 (mixed forests) in the “apples to apples” comparison, as the absolute RMS differences between PhenoCam and MODIS were actually no larger for IGBP class 5 than for IGBP class 1 (evergreen needleleaf forests) (Supplementary Table 2).

For GR sites, and during both the greenness rising and greenness falling phases, the correlation of PhenoCam and MODIS dates were as high as those for any other vegetation type (Fig. 2). And, for GR sites these correlation were as good for “apples to oranges” as for “apples to apples” comparisons (Table 2). In both cases, Deming regression slopes were not significantly different from 1.0 during either greenness rising or greenness falling phases (Table 2). However, the regression distance *d* tended to be marginally smaller for “apples to apples” comparisons than “apples to oranges” comparisons (Table 2).

**Figure 3:**
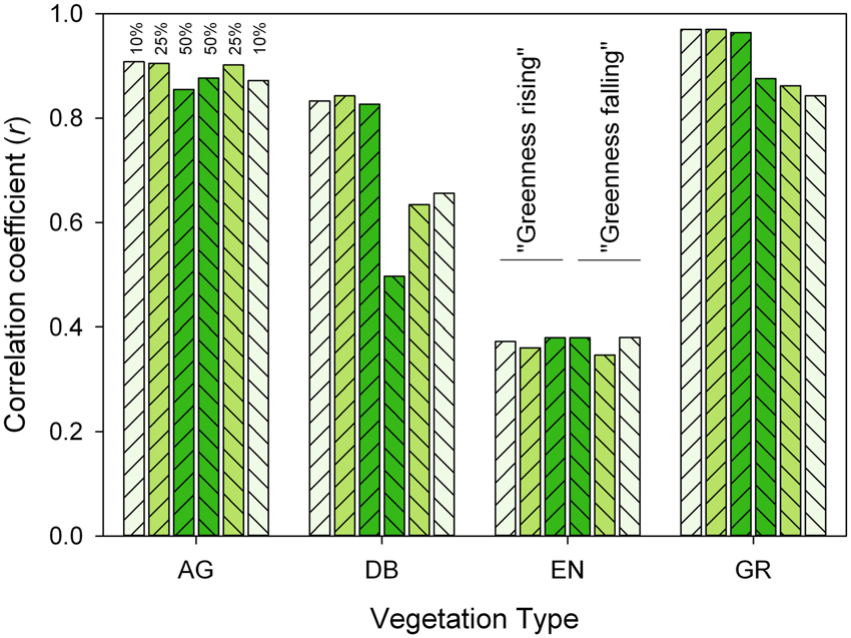
Correlation coefficient between phenological transition dates derived from PhenoCam digital camera imagery and from MODIS satellite remote sensing. Results are separated according to vegetation type (AG = agriculture; DB = deciduous broadleaf forest; EN = evergreen needleleaf forest; GR = grassland). Progressively darker shades of green are used to designate transition dates corresponding to 10%, 25%, and 50% of the seasonal amplitude in vegetation greenness. Patterning is used to distinguish greenness rising (rising left to right) and greenness falling (falling left to right) phases of the seasonal cycle.

The above results were conducted using the 10% amplitude threshold date as our phenological metric for the PhenoCam data. We initially selected the 10% threshold because it minimized the overall bias between PhenoCam transition dates and MODIS transition dates. But, for each ROI vegetation type, and for both greenness rising and greenness falling phases, we found that the results described above were not particularly sensitive to this choice (Fig. 3). For example, for AG sites, correlations were similarly strong (*r* ≈ 0.90 for both phases) regardless of whether 10%, 25% or 50% amplitude threshold dates were used. For DB, correlations were equally strong for all thresholds during the greenness rising phase, but the 10% threshold date generally performed marginally better than either the 25% or 50% threshold dates during the greenness falling phase. For EN, the agreement between PhenoCam and MODIS was generally poor for the 10% threshold date, as described above, and neither the 25% nor the 50% threshold dates substantially improved the correlation statistics (Fig. 3).

## Discussion

Our analysis has shown a generally high level of agreement between phenological transition dates derived from near-surface PhenoCam imagery and from MODIS satellite remote sensing. Relative to other studies that compared transition dates derived from MODIS data with ground observations recorded by citizen scientists^29,30^, our analysis shows as good or better agreement between what is seen from space and what is happening on the ground. The inherent subjectivity of ground observers, and the ability of PhenoCam imagery to integrate across the canopy, may be key factors contributing to these patterns.

We have drawn on a far more extensive set of PhenoCam-derived dates than have been used in previous phenological intercomparison studies^6,17,19,24,25^. This enhances our confidence in the robustness and generality of the results. However, we found that agreement varied according to vegetation type, with considerably better agreement for agricultural (AG), deciduous broadleaf (DB) forest, and grassland (GR) sites compared to evergreen needleleaf (EN) forest sites. We now use a case study approach to investigate and discuss the potential causes of varying levels of agreement between PhenoCam and MODIS. Landscape heterogeneity and a mismatch between the camera field of view and the associated MODIS pixel are recurring themes that will be discussed in the context of data from specific camera sites.

**Figure 4:**
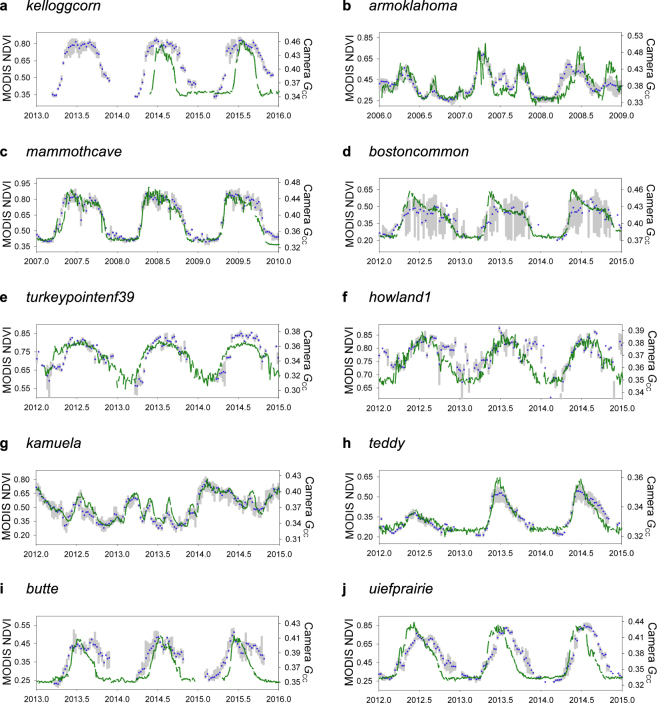
Sample time series showing agreement between satellite and near-surface remote sensing of vegetation phenology. Green lines indicate the Green Chromatic Coordinate (*G*_CC_) calculated from PhenoCam imagery (see Eq. 1). Grey bars indicate the spatial variability (range) in 500 m MODIS NDVI (normalized difference vegetation index) across a 3 × 3 pixel window centered on the camera’s location, with blue dots indicating the mean value. (**a**,**b**) Agricultural (AG) sites; (**c**,**d**) Deciduous Broadleaf (DB) forest sites; (**e**,**f**) Evergreen Needleleaf (EN) forest sites; (**g**–**j**) Grassland (GR) sites.

For PhenoCam AG sites, landscape heterogeneity appears to be a key issue. At the Kellogg Biological Station (*kelloggcorn*) site in Michigan, the PhenoCam data clearly resolve a well-defined seasonal cycle that starts later and ends earlier than would be inferred from the MODIS data (Fig. 4a). But, inspection of high-resolution aerial imagery (Google Earth) indicates the presence of deciduous forest stands to the east and south, which if included in the same MODIS pixel would potentially lead to the observed bias: trees within the camera field of view (but outside of the analyzed ROI) are green earlier in spring, and stay green later in autumn, than the corn field being monitored. Landscape heterogeneity would be less of an issue with finer-resolution satellite imagery, because pixels with mixed vegetation types would be less likely. Methods to estimate vegetation phenology from Landsat imagery, which is available at 30 m, have been recently developed and validated using PhenoCam data^24,31^. However, the 16-day revisit interval of Landsat 8 is a major limitation, and precludes phenological retrievals in many years^31^. Newer satellite platforms, offering both higher spatial and temporal resolution, such as the European Space Agency’s Sentinel-2 mission (every 5 days at 10 m resolution), have the potential to produce dramatically better characterization of vegetation phenology in heterogeneous landscapes. And, ongoing efforts to harmonize data from Landsat and Sentinel-2 will result in 30 m data at a temporal resolution of 2–3 days, offering substantial improvement over Landsat-only phenological retrievals.

At another AG site, the Atmospheric Radiation Measurement (*armoklahoma*) site in Oklahoma, the agreement between PhenoCam and MODIS is reasonably good through 2006 and 2007 but the earlier green-up seen by MODIS in 2008 is obvious (Fig. 4b). This leads to a 40-day difference between the MODIS onset of greenness date and PhenoCam greenness rising 10% threshold date. As at the *kelloggcorn* site, the PhenoCam at *armoklahoma* is focused on a relatively small field that is situated within a heterogeneous matrix of agricultural fields all subject to different planting and harvesting schedules, again suggesting that finer-resolution satellite imagery would result in improved agreement. On top of this, multiple vegetation cycles and sometimes abrupt changes in surface properties as a result of harvesting and tilling may combine with differing methods in transition date extraction^17^ to yield poor agreement.

Although DB forests in eastern North America are typically comprised of a diverse mix of species, stands are commonly extensive, stretching for kilometers across the landscape. Thus, fine-scale heterogeneity is offset by coarse-scale homogeneity^32^. By permitting integration across crowns of various species that make up the canopy, PhenoCam imagery effectively minimizes the impact of fine-scale heterogeneity on the derived phenological transition dates^33^. For example, at the Mammoth Cave National Park (*mammothcave*) site in Kentucky, where various oak (*Quercus* spp.) and hickory (*Carya* spp.) species dominate, and canopy cover is nearly continuous for 10 km in all directions, the coherence between PhenoCam GCC and MODIS NDVI is generally very good, particularly in springtime (Fig. 4c). It can be seen, however, that *G*_CC_ declines in autumn somewhat in advance of NDVI. This is likely because declines in *G*_CC_ are associated with changing leaf color in autumn, rather than actual shedding of leaves, which is what drives autumn declines in NDVI^34,35^. Thus, we believe these differences result predominantly from the differences in seasonality of *G*_CC_ and NDVI. However, saturation of NDVI at relatively low leaf area index values may also contribute to the observed pattern. Interestingly, in spring 2007 the initial rise in *G*_CC_ from 0.32 to 0.40, followed by a decline to 0.36, and then a secondary rise to a seasonal maximum of 0.44, is the result of the spring frost event following unusually early spring leaf-out at *mammothcave*. The frost event was seen across the southeastern US^36^. This event is not so clearly visible in the 16-day MODIS data, although the somewhat delayed increase in NDVI is consistent with frost damage.

At the Boston Common (*bostoncommon)* site in Massachusetts, the PhenoCam is directed at trees in a relatively large (1 km × 1 km) urban park^37^, which is bounded on all sides by a dense mix of residential and commercial development. The seasonality of the trees within the PhenoCam field of view is evident, and approximately tracks the upper envelope of the 500 m MODIS data for a 3 × 3 pixel window around the camera location (Fig. 4d). However, for the center pixel of this 3 × 3 window, the agreement between PhenoCam and MODIS dates is generally poor, presumably because this particular pixel is dominated by the built environment. Thus, as with the AG sites, there are instances where the resolution of MODIS satellite imagery hinders the agreement between near-surface and satellite remote sensing for DB sites. Additionally, at some DB sites, understory vegetation – which is often green earlier in spring and later in autumn than the overstory – may play a role in the earlier greenup onset (bias of 9 days early; Table 2) and later dormancy onset dates (bias of 15 days late; Table 2) observed by MODIS relative to PhenoCam^38,39^. At other sites, this effect may be negligible.

Because they retain foliage year-round, the seasonal changes in surface reflectance properties are generally smaller for EN forests than they are for DB forests, and hence these changes are harder to detect. In EN forests, changes in surface properties are driven by both changes in the pigmentation of existing foliage as well as the development of new foliage and senescence of old foliage^35,40^, complicating interpretation of derived phenophase transitions. And, while seasonal variation in evergreen conifer *G*_CC_ has been shown to correlate with changes in canopy photosynthesis^33,40^, the decoupling of conifer *G*_CC_ and NDVI has been noted previously^35,41^, which makes the poor agreement between PhenoCam and MODIS phenological transition dates for EN sites (Table 2, Fig. 3c,d) not particularly surprising. Even in the best of cases, such as the Turkey Point (*turkeypointenf39*) site in Ontario, where the seasonal cycles in both *G*_CC_ and NDVI are well-defined, it is clear that *G*_CC_ is ramping up ahead of NDVI in spring (Fig. 4e). At the Howland Forest Main Tower (*howland1*) site in Maine, agreement is hampered by the weakly defined and noisy seasonal dynamics of MODIS NDVI (Fig. 4f). For some evergreen sites, we found that the seasonality of EVI (the enhanced vegetation index)^42^ was in better agreement (*windriver*), but in other cases worse agreement (*niwot2*) with *G*_CC_. Since 2012, all newly deployed PhenoCams (over 200 to date) have been recording both visible and visible + near-infrared imagery, from which “camera NDVI” (and by extension “camera EVI”) can be calculated^41^. However, one previous study found that for EN forests, transition dates derived from camera NDVI were at best in only marginally better agreement than MODIS-derived transition dates^35^. Other vegetation indices might still be developed which would consistently provide a better characterization of seasonal changes in evergreen conifer canopies.

For grasslands, the overall agreement between PhenoCam and MODIS is very good, at least in extensive and homogeneous landscapes. For example, multiple seasonal cycles—associated with moisture pulses that are irregular in timing and duration^9^—are well-resolved at the Parker Ranch (*kamuela*) site in Hawaii (Fig. 4g), a mixed C_3_/C_4_ grassland which extends, largely uninterrupted, for 10+ km towards the base of the Mauna Kea volcano. However, despite the similarity of the *G*_CC_ and MODIS time series, we found poor agreement between the derived transition dates, with differences of three weeks or more during the greenness rising phase. In cases like this, a more flexible curve-fitting approach (e.g. the spline-based method used for PhenoCam data) might yield better agreement. Additionally, the existing MODIS algorithm resolves at most two seasonal cycles per year, while in some years at this site more than three green-up cycles are observed.

At the Teddy Roosevelt National Park (*teddy*) site in North Dakota, which is also a fairly extensive and homogeneous grassland landscape, both PhenoCam and MODIS show strong reductions in vegetation greenness in response to the summer 2012 drought^43^, and similar capacity to detect recovery in 2013 and 2014 (Fig. 4h). However, because of the small amplitude of greenness change in 2012, phenological transition dates were not retrieved from the PhenoCam *G*_CC_ time series. The minimum amplitude requirement in the PhenoCam transition date procedure is intentionally designed to minimize false positives that do not actually correspond to real phenological transitions on the ground. However, a trade-off is that important phenological anomalies—e.g. as would be associated with extreme or unusual weather events—may be missed.

For other GR sites, landscape heterogeneity is more of an issue. For example, the Continental Divide (*butte*) site in Montana, although classified as grassland (IGBP class 10), is in fact situated in a heterogeneous environment, as open grassland is mixed with irrigated residential lawns and riparian shrubs all within approximately 100 m of the camera location. Because this variation occurs at relatively small spatial scales (less than the 500 m size of MODIS pixels), it is not obviously visible in the 3 × 3 pixel window around the camera location, although the poor agreement between PhenoCam *G*_CC_ and MODIS NDVI is clear (Fig. 4i). During spring, MODIS greenup onset dates are more than a month earlier than those from the *butte* PhenoCam, while during autumn MODIS dormancy onset dates are more than a month later. Likewise, the University of Illinois Energy Farm’s Restored Prairie (*uiefprairie*) site, consisting of native grasses and forbs, is located within an agricultural matrix including adjacent fields of corn (*Zea mays*) and two switchgrass species (*Panicum virgatum*, *Miscanthus* x *giganteus*). Each of the four fields is observed by a different camera, but the prairie camera and the *P*. *virgatum* camera (*uiefswitchgrass* site) are mounted on the same mast. PhenoCam locations are defined by the location of the camera, and not the field of view of the camera. The resulting geolocation error is compounded by the heterogeneity of the vegetation. Consequently, the agreement between PhenoCam and MODIS is poor for *uiefprairie*, particularly in autumn (Fig. 4j). Efforts to geolocate the ROIs, rather than the camera itself could potentially improve agreement between dates derived from near-surface and satellite imagery, especially in heterogeneous landscapes. One approach would be to integrate information about the camera’s position (azimuth and angle of inclination, as well as height above ground) together with high-resolution Google Earth imagery to create an approximate projection of the camera field of view onto the landscape. At the same time, the representativeness of this projected ROI could be evaluated in the context of the broader landscape using the same Google Earth imagery.

Our analysis also indicated potentially poor agreement between PhenoCam and MODIS transition dates for tundra and wetland sites (Supplementary Table 2). Based on visual inspection of PhenoCam imagery, it appears that the poor mismatch for tundra sites may have partially resulted from our failure to distinguish snowy images from snow-free images: in some instances, for example, the identified greenness rising transition dates occurred when much of the landscape was still snow-covered. Ongoing work using deep learning methods to identify snow-contaminated PhenoCam images should help to improve transition date retrievals for cold, high-latitude sites. Also, however, the MODIS retrievals for some tundra sites (mid-late July for onset of greenness) would also appear to be suspect, and worthy of further investigation.

The above discussion has identified four key issues: (1) the limitations of coarse resolution of MODIS satellite imagery, particularly in heterogeneous landscapes, (2) the need for a more flexible approach to identify phenological transition dates from MODIS imagery, particularly in highly dynamic ecosystems; (3) the need for alternative vegetation indices that better characterize evergreen vegetation; and (4) the importance of more careful geolocation of PhenoCam data, and rigorous evaluation of whether the PhenoCam ROI is representative of the broader landscape. Resolving these issues will greatly enhance efforts to integrate phenological data across scales, and to apply a multi-scale phenological perspective to resource management and decision-making.

Repeat photography has long been used to track environmental change occurring on decadal time scales^44,45^. Applications of PhenoCam data go beyond phenology: the high frequency of PhenoCam imagery allows precise identification of the onset and duration of disturbance events, even months or years after these events have occurred. This has potential applications for both environmental monitoring and management^9^. Notable events observed in PhenoCam imagery include: extensive forest fire damage (*pasayten*, July 2014), controlled burn (*konza*, April 2014), forest tent caterpillar outbreak and defoliation (*canadaOA*, June 2016^46^), spring frost damage (*arbutuslake*, May 2010^47^), defoliation by Hurricane Irene (*woodshole*, August 2011), and progressively worsening forest mortality in California’s Sierra Nevada (*sequoia*, beginning in the summer of 2015). With fine-resolution PhenoCam imagery, these disturbance events can be visualized—and identified—in a way that is typically not possible with satellite data, or with near-surface radiometric instruments. PhenoCam data can uniquely provide context for interpretation of anomalies and outliers in time series of satellite vegetation indices. As the PhenoCam network continues to grow, the opportunities to use PhenoCam imagery, greenness time series, and phenological transition date data for evaluation and interpretation of remotely sensed data products, and linking from organisms to satellite pixels^32^, will only increase.

## Methods

Our analysis leverages data from PhenoCam and MODIS, as well as gridded land cover classification and climatological datasets. We describe these data first, and then summarize the analytical procedures employed.

### PhenoCam Data

We used the PhenoCam Dataset Version 1.0^27^, which is publicly available under the Creative Commons CC0 Public Domain Dedication. The processing routines by which the dataset was produced are fully described in the accompanying data descriptor^12^, but are briefly summarized here.

We used all camera sites (Supplementary Table 1) for which there were corresponding MODIS retrievals in the MCD12Q2 phenology product^3^. Camera images (minimally compressed, 3-layer JPEGs) were uploaded to the PhenoCam server as frequently as every 15 minutes (but typically every 30 minutes) from 4 am to 10 pm. For some older cameras, however, the upload frequency was only 1 image per day.

Image analysis consisted of several steps^12^. First, an appropriate “region of interest” (ROI) was defined, corresponding to the masked area within each digital image from which colour information would be extracted. We manually defined ROIs according the dominant vegetation type (or types, in the case where more than one vegetation type was clearly present) within each camera field of view. Second, the images were read sequentially, and the mean pixel value (digital number, DN) was determined across the ROI mask for each of the red, green and blue (RGB) color channels. This yielded an “RGB DN triplet” (R_DN_, G_DN_, B_DN_) for each image. Third, from the RGB triplet we calculated the Green Chromatic Coordinate (*G*_CC_; Eq. [1]), a simple measure of “canopy greenness” which has been used in numerous studies^16,18^ as a robust metric by which to characterize seasonal changes in the state of the canopy.

1 $${G}_{{\rm{cc}}}=\frac{{{\rm{G}}}_{{\rm{DN}}}}{{{\rm{R}}}_{{\rm{DN}}}+{{\rm{G}}}_{{\rm{DN}}}+{{\rm{B}}}_{{\rm{DN}}}}$$

*G*_cc_ was calculated for every image recorded when the sun was at least 10° above the horizon, provided that images were neither too dark nor too bright^12^. However, the resulting data density is excessive from the point of view of detecting seasonal changes. Here we use *G*_cc_ statistics calculated over a three-day moving window as a compromise between higher temporal resolution and improved noise reduction. The 90^th^ percentile value of *G*_cc_ (*G*_cc__90), calculated over this three-day moving window, has been shown to be generally effective for minimizing day-to-day variation due to weather (clouds, fog, aerosols) and illumination geometry^16^.

Aggregation to a three-day product was subsequently followed by outlier detection based on deviations from an optimally flexible smoothing spline, with the degree of smoothing identified using Akaike’s Information Criterion^12^. After outlier removal, the spline was re-fit and used to extract phenological transition dates from the *G*_cc_ time series. We applied the Pruned Exact Linear Time (PELT) method^48^ to parse the “greenness rising” and “greenness falling” phases from each spline. We calculated the seasonal amplitude of *G*_cc_ during each phase, and then identified the dates when the spline reached 10%, 25%, and 50% of the amplitude. Transition date uncertainties were estimated based on the uncertainty around the smoothing spline (90% confidence). The average uncertainty in estimated transition dates was about ±5 days.

Although successfully applied at many sites^16^, the *G*_cc__90 method has not been exhaustively validated across the entire PhenoCam data set. Thus, we determined the “best” *G*_cc_ time series for each (camera site) × (ROI) combination by identifying which of the *G*_cc_ time series (mean, 50^th^, 75^th^, and 90^th^ percentile values) had the lowest residual variance around the spline. We denoted this *G*_cc__best. Of the almost 200 (camera site) × (ROI) combinations in the data set, we found that in 45% of cases *G*_cc__mean was best, while *G*_cc__90 and *G*_cc__70 were each best in 20% of cases, and *G*_cc__50 was best in only 15% of cases. Therefore, contrary to previous results^16^, it appears that the *G*_cc__90 approach is not universally the optimal method. However, we found that transition dates derived from *G*_cc__best were extremely similar to those extracted from *G*_cc__90, pointing to the relative insensitivity of our results to the specifics of the *G*_cc_ time series processing. For example, for the greenness rising phase, the mean difference between dates derived from *G*_cc__best and *G*_cc__90 was 0.1 ± 4.7 d (with 90% of all differences in the range from −4 to +4 d) for the 10% amplitude threshold. Similarly, during the greenness falling phase, the mean difference between dates was −0.1 ± 8.0 d (with 90% of all differences in the range from −7 to +4 d) for the 10% amplitude threshold. Thus, while our analysis focuses on results derived from *G*_cc__best, the conclusions reached are relatively insensitive to this choice.

### MODIS data

For comparison with PhenoCam data, we assembled two datasets based on 500 m MODIS remote sensing imagery. First, we extracted the MCD12Q2 transition dates^3^ (calculated from the MCD43A4 NBAR EVI [enhanced vegetation index] product^49^) for each camera location, and paired these with the corresponding PhenoCam transition dates. MODIS dates were pulled from the Google Earth Engine (GEE) servers using the GEE subset tool^50^. Here, “corresponding” is defined to mean that *G*_cc_ and NDVI were moving in the same direction (“rising” or “falling”), and the absolute difference (in days) between MODIS and PhenoCam transition dates was less than 90 days.

To investigate the overall coherence between seasonal trajectories between PhenoCam *G*_cc_ and MODIS NDVI, we downloaded MCD43A4 Band 1 (620–670 nm) and Band 2 (841–876 nm) nadir reflectances (again at 500 m resolution) using the GEE subset tool. From these we calculated NDVI as:

2 $${\rm{NDVI}}=\frac{{\rm{Band}}\,2-{\rm{Band}}\,1}{{\rm{Band}}\,1+{\rm{Band}}\,2}$$

In addition to the MODIS pixel centered on the PhenoCam location, we included reflectance data from a 3 × 3 pixel window centered on the camera location as a means of examining coarse-scale spatial variation and landscape heterogeneity. Data from this 9-pixel window were used to calculate the range (maximum and minimum NDVI) shown in Fig. 4.

### Land cover classification and climatological data

PhenoCam site landcover was evaluated using the land cover classification scheme of the International Geosphere-Biosphere Programme, as derived from MODIS remote sensing at 500 m resolution^51,52^ (http://glcf.umd.edu/data/lc/). We used a majority value (excluding masked grid cells) for the 3 × 3 pixel window centered on the camera location.

Mean annual temperature (°C) and mean annual precipitation (mm) for each PhenoCam site were obtained from the WorldClim^28^ (http://worldclim.org/) database. These gridded climatological data are produced at a spatial resolution of approximately 1 km^2^.

### Statistical analysis

In our analysis of the agreement between PhenoCam and MODIS dates, we distinguished between “apples to apples” comparisons (similar vegetation types) and “apples to oranges” comparisons (dissimilar vegetation types). Our assignment of “apples” and “oranges” was based primarily on vegetation stature, leaf habit, and management activity. For example, for AG sites we considered IGBP types 12 (croplands) and 14 (cropland/natural vegetation mosaic) to be “apples”, but IGBP type 5 (mixed forest) to be an “orange”. Likewise, for DB sites we considered IGBP types 4 (deciduous broadleaf forest) and 5 (mixed forest) to be “apples”, but IGBP type 1 (evergreen needleleaf forest) to be an “orange”. In Supplementary Table 2, we report the RMS difference between PhenoCam and MODIS dates, tabulating by PhenoCam vegetation type for each separate MODIS land cover type. In Table 2, we present additional statistics, aggregating these results for “apples” and “oranges” comparisons.

We used Deming regression (commonly used in clinical chemistry to test whether two analytical methods yield comparable results^53^) to further evaluate the agreement between PhenoCam and MODIS dates. Specifically, we investigate whether the slope of the line relating PhenoCam and MODIS dates has a slope that is significantly different from 0 but not significantly different from 1, and we use the distance, *d*, between the data and the regression line as an overall regression error metric.

Deming regression accounts for measurement errors in both *x* and *y* variables, and for this reason is preferable to ordinary least squares which assumes that *x* is measured perfectly and only *y* is subject to error. In Deming regression, the ratio of the error variances is given by $\lambda ={\sigma }_{x}^{2}/{\sigma }_{y}^{2}$, and when the error variances are assumed equal (i.e. $\lambda$ = 1), Deming regression is equivalent to orthogonal regression, in which the perpendicular distance, *d*, from each data point to the regression line (intercept *b*_0_, slope *b*_1_) is minimized:

3 $$d=\frac{{(y-({b}_{0}+{b}_{1}x))}^{2}}{1+{({b}_{1})}^{2}}$$

Regression analyses were conducted in SAS 9.1 using *PROC NLP* for the $\lambda =1$ case, with sensitivity analyses for other values of *λ* conducted using the *Deming_Linnet* macro for SAS^54^ and the Deming regression package in SigmaPlot 12.5.

Based on previous analysis of the statistical uncertainty in phenological transition dates derived from PhenoCam and MODIS^17^, the most plausible range for $\lambda$ is between 0.25 and 1.0. We report results (Table 2) assuming $\lambda =1$, but we also repeated the analysis using $\lambda =[0.25,0.5,1,2,4]$. In general, although higher regression slope estimates were obtained when we specified a higher value of *λ*, conclusions about whether *b*_1_ was significantly different from 0 or 1 were not affected. The RMS values of *d* varied by less than 20% depending on *λ*, and Pearson correlation coefficients are insensitive to *λ*.

## Electronic supplementary material

Supplementary Information
